# Robot-assisted laparoscopic surgery for treatment of urinary tract stones in children: report of a multicenter international experience

**DOI:** 10.1007/s00240-021-01271-5

**Published:** 2021-05-16

**Authors:** Ciro Esposito, Lorenzo Masieri, Thomas Blanc, Thomas Lendvay, Maria Escolino

**Affiliations:** 1 Division of Pediatric Surgery, Federico II University of Naples, Via Pansini 5, 80131 Naples, Italy; 2 Division of Pediatric Urology, Meyer Children Hospital, Florence, Italy; 3 Division of Pediatric Urology, Hôpital Necker-Enfants Malades, Paris, France; 4 Division of Pediatric Urology, Seattle Childrens Hospital, Seattle, WA US

**Keywords:** Stones, Robot, Pyeloplasty, Children, Lithotomy, Nephroscopy

## Abstract

This study aimed to report a multi-institutional experience with robot-assisted laparoscopic surgery (RALS) for treatment of urinary tract stones in children. The medical records of 15 patients (12 boys), who underwent RALS for urolithiasis in 4 international centers of pediatric urology over a 5-year period, were retrospectively collected. The median patient age was 8.5 years (range 4–15). Eleven/fifteen patients (73.3%) had concurrent uretero–pelvic junction obstruction (UPJO) and 2/15 patients (13.3%) had neurogenic bladder. Stones were in the renal pelvis in 8/15 (53.3%), in the lower pole in 3/15 (20%), in the bladder in 2/15 (13.3%), and in multiple locations in 2/15 (13.3%). One patient (6.6%) had bilateral multiple kidney stones. The median stone size was 10.8 mm (range 2–30) in upper tract location and 27 mm (range 21–33) into the bladder. Eleven patients with concomitant UPJO underwent simultaneous robot-assisted pyelolithotomy and pyeloplasty in 12 kidney units. Two patients with isolated staghorn stones received robot-assisted pyelolithotomy. Robot-assisted cystolithotomy was performed in two patients with bladder stones. The median operative time was 131.8 min (range 60–240). The stone-free rate was 80% following initial surgery and 100% after secondary treatment. Clavien 2 complications (hematuria, infections) were recorded in 5/15 patients (33.3%). Three/fifteen patients (20%) with residual renal stones were successfully treated using ureterorenoscopy (Clavien 3b). RALS was a feasible, safe and effective treatment option for pediatric urolithiasis in selected cases such as large bladder stones, bilateral kidney stones, staghorn stones or concomitant anomalies such as UPJO requiring simultaneous pyeloplasty.

**Supplementary Information:**

The online version contains supplementary material available at 10.1007/s00240-021-01271-5.

## Introduction

The incidence of pediatric urolithiasis is rapidly increasing worldwide [1]. The main treatment options for urinary tract stones have been similar as those used in the adult population and included ureterorenoscopy (URS), retrograde intra-renal surgery (RIRS), extracorporeal shock wave lithotripsy (ESWL) and percutaneous nephrolithotomy (PCNL) [2–4]. However, these endo-urological techniques may not be adequate in patients presenting large impacted pelvic and ureteric stones, staghorn stones, an anomalous collecting system, or a synchronous pathology such as uretero–pelvic junction obstruction (UPJO), which should be preferably treated with the renal stones in a single surgical session [5]. These selected clinical conditions are better managed using an open or conventional laparoscopic approach [6, 7]. This is also supported by the European Association of Urology (EAU) guidelines for the management of urinary stone disease in children, stating that open or laparoscopic approach may be inevitable in such situations [8]. However, the morbidity of open surgery can be significant [9], whereas drawbacks of laparoscopic stone surgery include challenges with ureteral stenting, limited dissection and intra-corporeal suturing, as well as increased risk of complications such as urinary leakage [10].

More recently, the advent of robotic platform led to overcome many of the technical disadvantages of laparoscopy providing a three-dimensional view of the operative field, a precise dissection and easy suturing thanks to the 7° of movement of the robotic arms and excellent ergonomics [11, 12].

In the adult population, robot-assisted laparoscopic surgery (RALS) has been successfully employed for the treatment of renal stones during concomitant treatment of UPJO and for the primary treatment of staghorn stones [13, 14]. Robot-assisted pyelolithotomy, robot-assisted ureterolithotomy and robot-assisted flexible URS are currently part of the adult urologist’s armamentarium for treatment of large-volume stones and are very useful in conditions requiring simultaneous reconstruction [15, 16].

However, there is still limited evidence regarding the role of RALS in the management of pediatric urolithiasis [8].

This study aimed to report a multi-institutional international experience with RALS for treatment of urinary tract stones in children.

## Materials and methods

The medical records of 15 patients (12 boys and 3 girls) affected by urolithiasis, who underwent RALS in 4 international centers of pediatric urology over a 5-year period (January 2015–January 2020), were retrospectively collected and analyzed.

Pre-operative work-up included renal ultrasound (US), Kidney, Ureter, and Bladder (KUB) X-ray and/or computerized tomography (CT). A diuretic MAG3 renal scan was also performed in all patients with pre-operative suspicion of UPJO. RALS in our series was restricted to patients older than 1 year of age and with a body weight higher than 12 kg. All the surgical procedures were performed by one senior surgeon in each participating center, who had > 15 years of experience in laparoscopy and > 3 years of experience in robotics. Follow-up included clinical evaluation and radiographic imaging (KUB, renal US) at 1–3–6–12 months postoperatively and thereafter annually.

The primary outcome of the study was the stone-free rate. Successful stone-free outcome was defined as no documented stone fragments on postoperative radiographic imaging (KUB, renal US). If a concomitant UPJO existed, surgical success was defined by improvement of hydronephrosis with anterior–posterior pelvic diameter (APD) < 10 mm on US and relief of obstruction on MAG3 renal scan, defined by the presence of ureteric excretion at least < 10 min.

Secondary outcome parameters included operative time, analgesic requirement, length of hospital stay, conversions, intra- and postoperative complications, and re-operations. Operative time was defined as the total time spent into the operating room from the skin incisions (port placement) to completion of skin closure. Postoperative complications were classified according to the Clavien–Dindo grading system [17].

The authors collected data on demographics, symptoms at presentation, and pre-operative stone data, including size, location, and number of stones. The authors also collected data on stone composition, which was reported as the majority composition (> 50%), whereas any uric acid or struvite stones were classified as such.

The study received the appropriate Institute Review Board (IRB) approval at each participating center.

### Operative technique

#### Renal stones

**Fig. 1:**
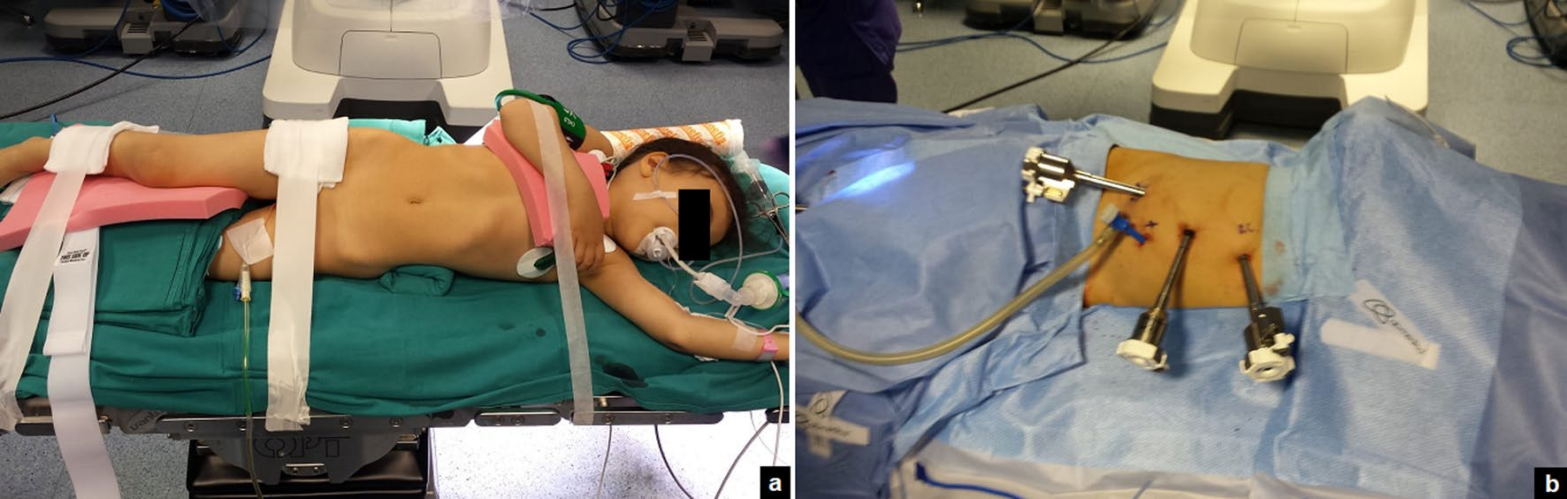
Patient (**a**) and ports (**b**) position in a simultaneous right pyelolithotomy and pyeloplasty

**Fig. 2:**
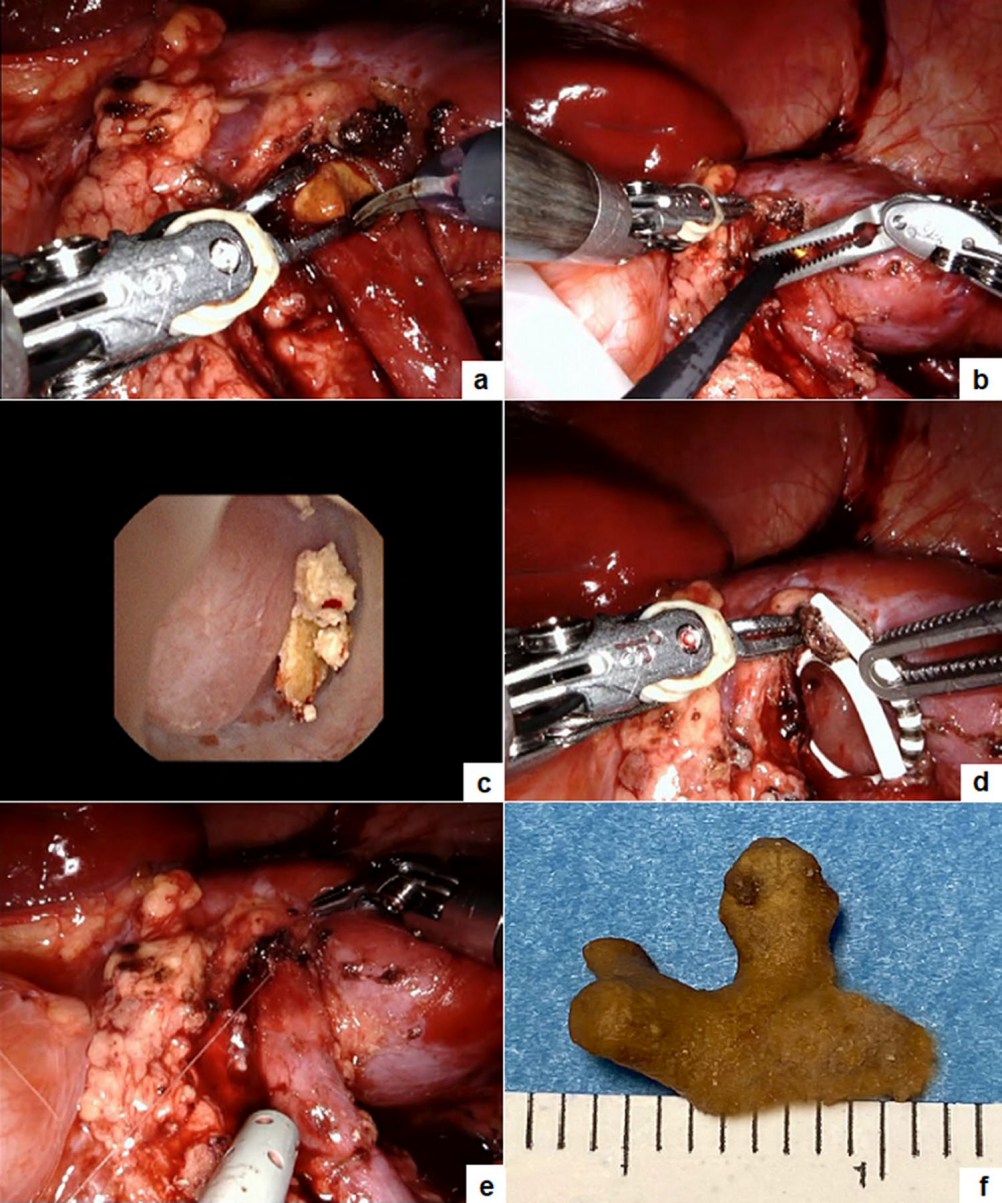
Operative steps of pyelolithotomy: the renal pelvis is opened and the stone is extracted using robotic graspers (**a**); the ureteroscope is introduced into the renal pelvis (**b**) and the residual fragments are removed under vision (**c**); a double J stent is inserted (**d**); the renal pelvis is closed using interrupted stitches (**e**); the stone is extracted (**f**)

After the induction of general anesthesia, the patient was rolled into a semilateral decubitus position rotating the operative side up by 45° axially using silicone pads underneath the patient (Fig. 1a). An age-appropriate bladder catheter was inserted pre-operatively using sterile precautions. Four ports were positioned (Fig. 1b): the first 8-mm robotic camera port was placed infra-umbilically using open Hasson technique; after induction of pneumoperitoneum, the two operative 8-mm robotic ports were placed under vision on the emiclavear line, one 2 cm under the subcostal arch and the other 3 cm above the inguinal ligament. Finally, the fourth 5-mm assistant port was positioned on the pararectal line, mean 7 cm caudal to the robotic camera port and the da Vinci Xi robot was docked, using a three-arm configuration. A 30° optic was used. The transperitoneal route was adopted in all cases. After incision of the Toldt line and the lowering of the colon, the renal pelvis was detected and incised with non-electrified scissors. The stones were identified in the renal pelvis and removed using robotic graspers. Individual calices were inspected using a flexible ureteroscope introduced through the assistant/robotic port. Identified stones were grasped with a flexible grasper or using a basket and extracted through the assistant/robotic port (Fig. 2). If the stones could not be directly removed via the assistant/robotic port, they were placed in a sterile glove finger introduced intra-corporeally through the assistant/robotic port. The glove was then removed through the umbilical port at the end of the procedure. The renal pelvis was gently washed with saline in order to remove all stone fragments. If a concomitant UPJO existed, it was repaired at this time. A dismembered Anderson–Hynes pyeloplasty was performed using 6-0 or 5-0 poliglecaprone 25 suture: two or three interrupted stitches were placed at each end of the spatulated ureter and the renal pelvis and a running suture completed the posterior wall of the ureteropelvic anastomosis. An indwelling double J stent was placed in the ureter in an anterograde fashion through the assistant port. The anterior wall of the ureteropelvic anastomosis was finally completed using a second running suture. The Toldt fascia was reconstructed with interrupted stitches and a 15 F abdominal drain was placed in some patients through the 5-mm assistant port and left in place for at least 48–72 h postoperatively. Trocars’ orifices were closed using resorbable sutures.

#### Bladder stones

The patient was placed in supine position with the legs apart, and the operating table was placed in 10° Trendelenburg position to encourage the bowel to fall out of the pelvis. An age-appropriate bladder catheter was inserted pre-operatively using sterile precautions. Four trocars were placed: one 8-mm robotic port was inserted in the umbilicus for the 0° robotic optic and other two 8-mm robotic ports were inserted at 7–9 cm apart from the camera port along the midclavicular line bilaterally. Finally, the fourth 5-mm assistant port was placed in the ipsilateral hypochondriac region. In children less than 20 kgs, the working ports were preferably placed at the level of the umbilicus to ensure a good distance to the target site. The robot was then docked over the patient’s feet. The bladder was suspended using two stay sutures that were introduced trans-abdominally. After filling the bladder with normal saline, a 3.5-cm longitudinal incision of the detrusor muscle was performed in the midline using monopolar scissors, till the mucosa was seen pouting out. The bladder mucosa was incised and the bladder cavity was inspected. Once visually identified the big stone, it was grasped using the robotic grasper and put into a retrieval bag that was extracted through the umbilical port at the end of the procedure. The bladder was gently flushed with normal saline in order to ensure removal of all stone fragments and the bladder wall was finally reconstructed using a two-layer running 3-0 polyglactin suture, involving the mucosa and thereafter the detrusor muscle. Trocars’ orifices were closed using resorbable sutures. No abdominal drain was placed at the end of the surgery, whereas an indwelling Foley catheter was left in the bladder.

Video 1 reproduced all the steps of the surgical technique.

## Results

**Fig. 3:**
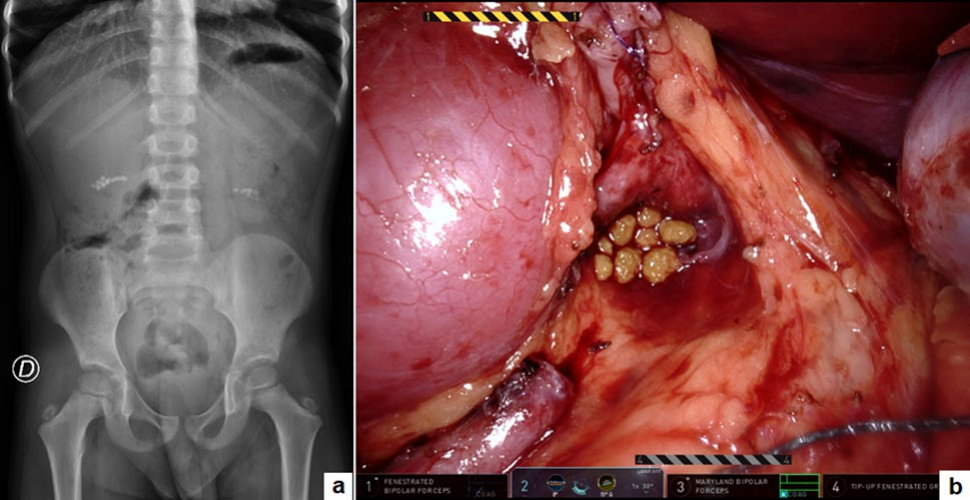
Bilateral multiple kidney stones: KUB (**a**) and intra-operative view (**b**)

**Fig. 4:**
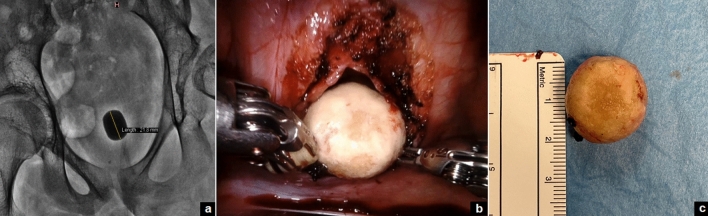
Large bladder stone: KUB (**a**); intra-operative view (**b**); ex vivo (**c**)

The median patient age at surgery was 8.5 years (range 4–15) and the median weight was 32.2 kg (range 14–55). Eleven/fifteen patients (73.3%) had a concurrent UPJO and 2/15 patients (13.3%) had neurogenic bladder and practiced clean intermittent catheterization (CIC) at 3-h intervals and oxybutynin. Most patients (13/15, 86.7%) were symptomatic with colicky flank pain, hematuria and urinary tract infections (UTIs). This was an incidental finding in two asymptomatic patients (13.3%). Stones were in the renal pelvis in 8/15 (53.3%), in the lower pole in 3/15 (20%), in the bladder in 2/15 (13.3%), and in multiple locations in 2/15 (13.3%). One patient (6.6%) had bilateral multiple stones in the renal pelvis/lower pole (Fig. 3). The median number of stones was 3 (range 1–15). The median stone size was 10.8 mm (range 2–30) in the upper tract location and 27 mm (range 21–33) into the bladder. Eleven patients with concomitant UPJO underwent simultaneous robot-assisted pyelolithotomy and pyeloplasty in 12 kidney units. Two patients with isolated staghorn stones received robot-assisted pyelolithotomy (Fig. 2). An intra-operative nephroscopy was also performed in all cases (Fig. 2). A robot-assisted cystolithotomy was performed in two patients with bladder stones (Fig. 4). One of these patients had previous failed cystoscopy. All the procedures were accomplished robotically with no need for conversion or intra-operative complications. The median operative time was 131.8 min (range 60–240) and the median hospital stay was 2.8 days (range 1–4). The median bladder catheter duration was 2 days (range 1–4) after pyelolithotomy and 15.5 days (range 10–21) after cystolithotomy. The double J stent was removed via cystoscopy under short duration anesthesia at median 21 days (range 18–31) postoperatively.

The median follow-up length was 23.2 months (range 3–61). The stone-free rate following initial surgery was 80%. The overall surgical success rate in patients who underwent concomitant pyeloplasty was 100%, with significant improvement of both hydronephrosis at renal US and renal excretion at MAG3 renal scan. Regarding postoperative complications, Clavien 2 complications (hematuria, UTIs) were recorded in 5/15 patients (33.3%). Three/fifteen patients (20%) presented residual renal stones following robot-assisted pyelolithotomy (Clavien 3b) and were successfully treated using ureterorenoscopy. After secondary treatment, the final stone-free rate was 100%.

Stone composition was calcium oxalate in 7/15 (46.7%), calcium phosphate in 5/15 (33.3%), and struvite in 3/15 (20%).

Patient demographics and operative and postoperative data are reported in Tables 1 and 2.

**Table 1:** Patient demographics

Demographics		
Case number, *n*	15	
Gender (male), *n* (%)	12 (80%)	
Median age, years (range)	8.5 (4–15)	
Median weight, kgs (range)	32.2 (14–55)	
Associated anomalies		
Uretero–pelvic junction obstruction (UPJO), *n* (%)	11 (73%)	
Neurogenic bladder, *n* (%)	2 (13.3%)	
Other, *n* (%)	0	
Presentation symptoms		
Colicky flank pain, *n* (%)	6 (40%)	
Colicky flank pain, hematuria, *n* (%)	2 (13.3%)	
Urinary tract infections (UTIs), *n *(%)	5 (33.3%)	
Asymptomatic, incidental finding, *n* (%)	2 (13.3%)	
Stone location		
Renal pelvis, *n* (%)	8 (53.3%)	
Lower pole, *n* (%)	3 (20%)	
Renal pelvis and lower pole, *n* (%)	2 (13.3%)	
Bladder, *n* (%)	2 (13.3%)	
Affected kidney data (*n* = 13)		
Side		
Left, *n* (%)	4 (30.8%)	
Right, *n* (%)	8 (61.5%)	
Bilateral, *n* (%)	1 (7.7%)	
Total renal units, *n*	15	
Location		
Orthotopic, *n* (%)	13 (100%)	
Pelvic, horseshoe, *n* (%)	0	
Stone pre-operative data		
Median stone size, mm (range)	20.8 (3–30)	
Median number of stones, *n* (range)	3 (1–15)	
Multiple stones present, *n* (%)	5 (35.7%)	
Indications for RALS		
Concomitant pyeloplasty, *n* (%)	11 (73.3%)	
Large stone size, *n* (%)	7 (46.6%)	
Previous failed endourological procedure, *n* (%)	1 (6.6%)	

*RALS* robot-assisted laparoscopic surgery

**Table 2:** Operative and postoperative data

Operative and postoperative data	
Surgical procedure	
Anderson-hynes dismembered pyeloplasty + pyelolithotomy, *n*	11
Pyelolithotomy, *n*	2
Intra-operative nephroscopy, *n*	13
Cystolithotomy, *n*	2
Postoperative drainage	
Indwelling JJ stent, *n* (%)	13 (86.7%)
Abdominal drain, *n* (%)	6 (40%)
Indwelling bladder catheter, *n* (%)	15 (100%)
Median duration of JJ stent, days (range)	21 (18–31)
Median duration of bladder catheter after pyelolitothomy, days (range)	2 (1–4)
Median duration of bladder catheter after bladder lithotomy, days (range)	15.5 (10–21)
Median operative time, minutes (range)	131.8 (60–240)
Intra-operative complications, *n* (%)	0
Conversion, *n* (%)	0
Median analgesic requirement, hours (range)	24.5 (8–48)
Median hospital stay, days (range)	2.8 (1–4)
Stone Composition	
Calcium oxalate, *n* (%)	7 (46.7%)
Calcium phosphate, *n* (%)	5 (33.3%)
Struvite, *n* (%)	3 (20%)
Median follow-up length, months (range)	23.2 (3–54)
Stone-free rate	
Following initial surgery, *n* (%)	12 (80%)
Following secondary procedure, *n* (%)	15 (100%)
Residual stone management	
Ureteroscopy, *n* (%)	3 (20%)
Overall success rate of pyeloplasty*, n *(%)	11 (100%)
Degree of hydronephrosis at US	
Median pre-operative APD, mm (range)	28.4 (18–40)
Median postoperative APD, mm (range)	4.1 (0–8)
Renal drainage on Mag 3 renogram	
Before surgery, minutes (range)	44 (27–78)
After surgery, minutes (range)	7.7 (8–15)
Postoperative complications	
Hematuria, *n* (%)	3 (20%)—II grade Clavien
Urinary tract infections (UTIs), *n* (%)	2 (13.3%)—II grade Clavien
Residual stones, *n* (%)	3 (20%)—IIIb grade Clavien
Stricture of ureteropelvic anastomosis, *n* (%)	0
Other, *n* (%)	0

*APD* anterior–posterior pelvic diameter, *US* ultrasound

## Discussion

The treatment of urinary tract stones, especially large stone burdens, may be challenging in children and require multiple procedures [18]. The American Urological Association (AUA) and European Association of Urology (EAU) guidelines [8, 19, 20] for treatment of urinary tract stones in adults indicated that PCNL is the treatment of choice for kidney stones > 2 cm; whereas, flexible URS/RIRS and ESWL are the first-line therapy for stones < 2 cm. The same principles are also applied in children and adolescents [8]. However, multiple treatment sessions may be required to achieve stone clearance [18]. Furthermore, ESWL is not recommended in children with stone burdens larger than 25 mm [21]. Laparoscopic and robot-assisted approaches have been reported as viable treatment options in selected patients with large stones, anomalies of collecting system and complex stone burdens [7–10]. These indications were supported by a conspicuous evidence in the adult population [13–16, 22]. Obviously, RALS overcomes the potential technical challenges of the laparoscopic approach, related to intra-corporeal dissection and suturing, due to its demonstrated advantages of improved dexterity and three-dimensional visualization [11, 12]. However, there is still limited evidence regarding the role of RALS in the management of pediatric urolithiasis [8]. Analyzing the pediatric literature, the first study focused on the robot-assisted management of stone disease in the pediatric population was published in 2007 by Lee et al. [23], who reported a safe and effective use of robot-assisted pyelolithotomy in five adolescents with large stone burdens. The second and last published study confirmed that endoscopic-assisted robotic pyelolithotomy is a reasonable alternative to endourological and percutaneous approaches for selected pediatric patients with concomitant ureteropelvic junction obstruction and nephrolithiasis or with stones inaccessible by standard methods [24]. Considering the paucity of available data, we decided to organize a multicenter study to collect the experience of different international centers of pediatric urology with strong experience in this field. Based upon this multi-institutional experience, we would make some general considerations about this application of RALS.

First, the main indications for RALS in pediatric urolithiasis, as reported in our series, should include patients with large stones or bilateral stones; patients who underwent previous failed endourological procedures; and patients with concomitant anomaly of collecting system, such as ureteropelvic junction obstruction, requiring simultaneous reconstruction [8].

The first important indication for RALS is in patients with large stones or staghorn stones, when PCNL cannot be performed because of a lack of surgical expertise or other minimally invasive procedures fail [23–25]. In our series, one patient with neurologic bladder had a large bladder stone. After a failed attempt with cystoscopy, he was successfully treated using robot-assisted cystolithotomy, with no intra-operative technical challenges or postoperative complications. The only precaution was to leave an indwelling bladder catheter for 3 weeks postoperatively before restarting the CIC to avoid any potential injury on the bladder dome suture due to the self-catheterization. To our knowledge, this is the first report of robot-assisted cystolithotomy in the pediatric population.

Bilateral localization of kidney stones represents a further challenging scenario. Simultaneous bilateral robotic procedures on kidney have been already described, both in children and adults [14, 26, 27]. Frileich et al. [28] demonstrated that simultaneous bilateral robot-assisted laparoscopic pyeloplasty provided an effective method of managing patients with bilateral UPJO avoiding the burden and morbidity of performing stage surgeries. We reported the first pediatric case with bilateral multiple kidney stones and UPJO who was successfully treated with simultaneous bilateral robot-assisted pyelolithotomy and pyeloplasty. Use of RALS allowed us to accomplish the simultaneous surgery on both sides with minimal morbidity and avoiding the risks of multiple procedures [26].

Although the EAU guidelines recommended that robotic-assisted laparoscopic pyeloplasty and surgical treatment for stones should be performed in separate procedures [8, 29], our study demonstrated that simultaneous pyelolithotomy and pyeloplasty in a single surgical session was a safe and feasible option for patients with kidney stones and concomitant UPJO. Furthermore, it was highly effective with 100% success rate of the pyeloplasty, 80% stone-free rate following initial surgery and 100% stone-free rate following secondary procedure. Another important advantage of performing both surgeries concurrently was to avoid an additional anesthesia, reducing the potential negative effects reported by general anesthetics on the brain development of children undergoing multiple and long-lasting surgical procedures [8].

Second, we would report some tips and tricks of the technique to achieve high stone clearance rate. It is very useful to perform in all kidney stones an intra-operative nephroscopy through the pyelolithotomy incision to explore the entire pelvicalyceal system, regardless of stone location in the upper collecting system [8, 24]. We always adopted a flexible ureteroscope that was easily introduced through the assistant port or a robotic port after one of the arms was undocked. Use of flexible ureteroscope was very useful in case of multiple stones, staghorn stones or stones in a difficult location, such as a lower pole calyx, allowing to reposition the stone into a more convenient position or to use stone baskets to maximize the stone clearance [23, 30]. The stone was retrieved using the robotic graspers, if it was easily visible in the renal pelvis or, alternatively, using endoscopic graspers or baskets [9]. Another important trick was to gently flush the calices and the renal pelvis using normal saline at the end of the procedure to wash all cavities and remove all fragments. Furthermore, for removal of stones from the abdominal cavity, although they can be directly extracted through the assistant port or a robotic port [8], we advise to put all fragments into a retrieval bag or a homemade bag, using the index finger of a sterile glove, and extract it through the umbilical port. Using this trick, the potential risk of fragment loss into the peritoneal cavity is prevented.

Limitations of this study are its retrospective nature and the small number of patients included; however, this limitation is due to the low volume case in the pediatric population. For this reason, we collected the data from several pediatric centers with the efforts to achieve a larger case series. Furthermore, the rarity of this procedure makes a prospective study challenging.

In conclusion, our results showed that RALS was a feasible, safe and effective treatment option for pediatric urinary tract stones in selected cases such as complex urinary tract stones, large bladder stones, bilateral kidney stones, staghorn stones, prior failed endoscopic procedures or concomitant urinary anomalies such as UPJO requiring simultaneous pyeloplasty. The possibility to perform an intra-operative nephroscopy using flexible ureteroscope allowed to achieve a high stone-free rate.

## Supplementary Information

Below is the link to the electronic supplementary material.

Video 1: Operative steps of robot-assisted cystolithotomy (MP4 11767 KB)

## Data Availability

All data generated or analyzed during this study are included in this published article [and its supplementary information files].
